# Effect of Escalating Financial Incentive Rewards on Maintenance of Weight Loss: A Randomized Clinical Trial

**DOI:** 10.1001/jamanetworkopen.2019.14393

**Published:** 2019-11-01

**Authors:** William S. Yancy, Pamela A. Shaw, Catherine Reale, Victoria Hilbert, Jiali Yan, Jingsan Zhu, Andrea B. Troxel, Gary D. Foster, Kevin G. Volpp

**Affiliations:** 1 Duke University Diet and Fitness Center, Durham, North Carolina; 2 Department of Medicine, Duke University School of Medicine, Durham, North Carolina; 3 Center of Innovation to Accelerate Discovery and Practice Transformation, Department of Veterans Affairs, Durham, North Carolina; 4 Department of Biostatistics, Epidemiology and Informatics, Perelman School of Medicine, University of Pennsylvania, Philadelphia; 5 Leonard Davis Institute Center for Health Incentives and Behavioral Economics, Perelman School of Medicine, University of Pennsylvania, Philadelphia; 6 Department of Health Care Management, The Wharton School, University of Pennsylvania, Philadelphia; 7 Center for Clinical Epidemiology and Biostatistics, Perelman School of Medicine, University of Pennsylvania, Philadelphia; 8 Department of Medical Ethics and Health Policy, Perelman School of Medicine, University of Pennsylvania, Philadelphia; 9 Department of Medicine, Perelman School of Medicine, University of Pennsylvania, Philadelphia; 10 Department of Population Health, New York University School of Medicine, New York; 11 Department of Science, WW (formerly Weight Watchers), New York, New York; 12 Center for Obesity Research and Education, Temple University, Philadelphia, Pennsylvania; 13 Center for Weight and Eating Disorders, Perelman School of Medicine, University of Pennsylvania, Philadelphia; 14 Center for Health Equity Research and Promotion, Philadelphia Veterans Affairs Medical Center, Philadelphia, Pennsylvania

## Abstract

**Importance:**

Identifying effective strategies for treating obesity is a public health priority.

**Objective:**

To test an escalating lottery-based incentive tied to daily self-weighing for weight loss maintenance.

**Design, Setting, and Participants:**

This 2-phase, 2-arm randomized clinical trial enrolled participants aged 30 to 80 years with an initial body mass index (calculated as weight in kilograms divided by height in meters squared) of 30.0 to 45.0 who lost at least 5 kg during 4 to 6 months in a national online weight management program. Study investigators and outcomes assessors were blinded to participant groups. Data were collected and analyzed from May 23, 2016, through November 13, 2017, based on intention to treat.

**Interventions:**

All participants were advised to weigh themselves daily, with a goal of 6 or more days per week, and received text messaging feedback on their performance. Incentive group participants were eligible for a lottery-based incentive worth an expected value of $3.98 in week 1 that escalated by $0.43 each week they achieved their self-weighing goal during months 1 to 6 (phase 1), followed by no incentives during months 7 to 12 (phase 2).

**Main Outcomes and Measures:**

The primary outcome was weight change at the end of phase 1. Secondary outcomes included weight change at the end of phase 2 and changes in self-reported physical activity and eating behaviors.

**Results:**

A total of 258 participants (128 in the incentive group and 130 in the control group) had a mean (SD) age of 48.0 (10.5) years and mean (SD) body mass index of 32.1 (3.9); 225 (87.2%) were women; 235 (91.1%) were white; and 102 (39.5%) had an annual income of at least $100 000. Weight measurement was transmitted by 250 participants (96.9%) at 6 months and 227 (88.0%) at 12 months. The percentage of weeks that incentive and control participants achieved a mean self-weighing of at least 6 times was 85.3% vs 75.8%, respectively (*P* = .002) in phase 1 and 37.7% vs 50.2%, respectively (*P* = .009) in phase 2. Mean weight changes at the end of phase 1 were −1.1 (95% CI, −2.1 to −0.1) kg in the incentive group and −1.9 (95% CI, −2.9 to −0.8) kg in the control group, with a mean difference of 0.7 (95% CI, −0.7 to 2.2) kg (*P* = .30 for comparison). At the end of phase 2, mean weight changes were 0.2 (95% CI, −1.2 to 1.7) kg in the incentive group and −0.6 (95% CI, −2.0 to 0.8) kg in the control group, with a mean difference of 0.8 (95% CI, −1.2 to 2.8) kg (*P* = .41 for comparison).

**Conclusions and Relevance:**

Compared with an active control condition of weekly messages, escalating lottery-based incentives transiently increased rates of self-weighing but did not significantly enhance weight loss maintenance.

**Trial Registration:**

ClinicalTrials.gov identifier: NCT01900392

## Key Points

**Question:**

Does an escalating lottery-based incentive tied to daily self-weighing improve weight loss maintenance?

**Findings:**

In this randomized clinical trial that included 258 adults from an online weight management program, escalating lottery-based incentives transiently increased rates of self-weighing but did not significantly enhance mean weight loss maintenance compared with an active control at 6 months or after incentives had been removed for 6 months.

**Meaning:**

In this study, escalating lottery rewards for self-weighing did not result in significantly greater maintenance of weight loss compared with an active control group.

## Teaser

This randomized clinical trial assesses an escalating lottery-based incentive tied to daily self-weighing for weight loss maintenance among adults in an online weight management program.

## Introduction

Because of its high prevalence and association with multiple illnesses, obesity has become a leading cause of preventable death in the United States.^1^ Identifying effective strategies for treating obesity is a clinical challenge and a public health priority. Although a variety of approaches have been successful in achieving initial weight loss, maintenance of weight loss has proven much more difficult.

Research has shown that the frequency of self-weighing is important for prevention of weight gain and for weight loss.^2,3^ Financial incentives have been successful at producing behavior change, including weight loss, in a variety of settings and formats, with lottery-based incentives being an effective design. Financial incentives, however, have been studied infrequently in the context of weight loss maintenance. In a previous study enrolling participants who had already lost 5 kg in a commercial weight management program, direct and lottery-based incentives did not enhance weight outcomes beyond the control intervention.^4^ However, participants who self-weighed at least 6 days each week experienced better weight outcomes than those weighing less frequently. Given the difficulties of sustaining adherence over time during weight control efforts, we hypothesized that a reward that increased over time with persistent adherence to self-weighing at least 6 days each week would improve weight outcomes. Therefore, our primary aim was to assess the effectiveness of escalating lottery rewards, relative to a control condition, on maintenance of weight loss during the 6 months after an initial weight loss of at least 5 kg. Our secondary aim was to assess the durability of this effect after an additional 6 months without rewards.

## Methods

### Overview of Study Design

**Figure 1.**
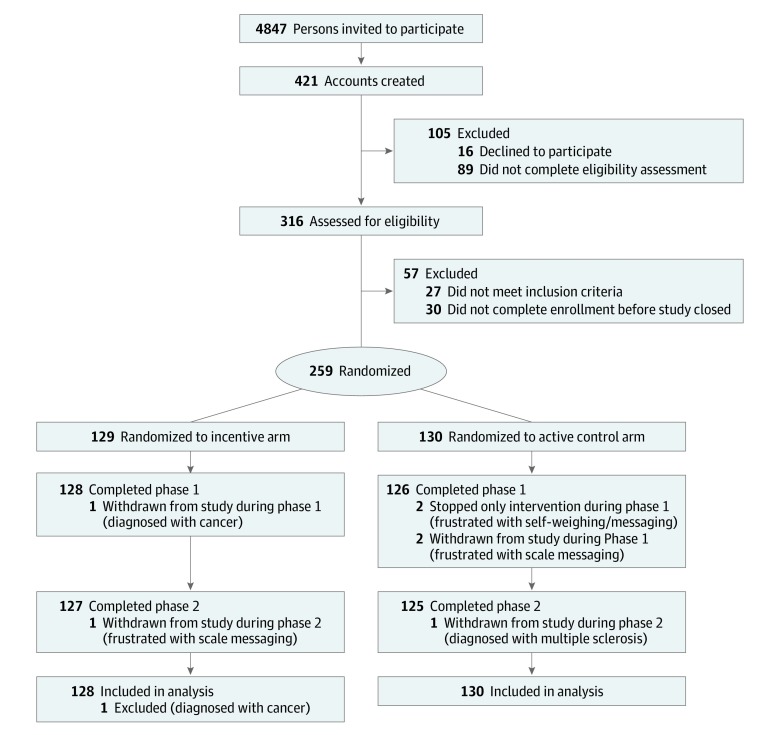
CONSORT Flow Diagram of Study Participants

The study was a 2-phase, 2-arm randomized clinical trial (trial protocol is available in Supplement 1) in which participants who lost at least 5 kg in a commercial weight management program were randomized in a 1:1 ratio to (1) daily self-weighing and weekly feedback (control group) or (2) daily self-weighing, weekly feedback, and a weekly, escalating, lottery-based financial incentive for 6 months (phase 1) (Figure 1). Participants in both groups were then observed without intervention for an additional 6 months (phase 2). Recruitment for the study began May 23, 2016, and ended November 13, 2016, when target sample size was reached, with the final participant completing the follow-up period on November 13, 2017. The study was approved by the institutional review boards of the University of Pennsylvania, Philadelphia, and Duke University, Durham, North Carolina. All patients provided written informed consent. This report follows the Consolidated Standards of Reporting Trials (CONSORT) reporting guideline for randomized clinical trials.^5^

### Participants and Setting

Participants were recruited by email from WW (formerly Weight Watchers) Digital to members who had opted in for communications from WW about research studies and met the following inclusion criteria: aged 30 to 80 years, body mass index (calculated as weight in kilograms divided by height in meters squared) of 30.0 to 45.0 before starting WW, documented weight loss of at least 5 kg in the previous 4 to 6 months, and stable health. Participants were enrolled in the study by a clinical research coordinator (C.R.). Potential participants were excluded for the following: substance abuse; bulimia nervosa or related behaviors; pregnancy or breastfeeding; medical contraindications to counseling about diet, physical activity, or weight reduction; unstable mental illness; positive screen findings for pathologic gambling^6^; or inability to read or complete forms in English. Enrollment, electronic written informed consent, and data collection occurred via the Penn Way to Health portal, a web-based infrastructure used to run behavioral economic intervention studies.^4,7^ Before signing the informed consent form electronically, the participant and clinical research coordinator were blinded to the potential group assignment. Participants were assigned a randomly generated identification number by the Way to Health platform and were randomized by a research coordinator (C.R.) using an electronic number generator through the Way to Health platform after consent was obtained and eligibility was confirmed. Investigators and outcomes assessors were blinded to participants’ group assignments.

### Interventions

Participants in both groups were asked to self-weigh on a provided digital, internet-enabled wireless scale (Withings) at least 6 of 7 days each week and received weekly tips regarding nutrition, physical activity, and self-weighing. The scale wirelessly transmitted their weight data to the Way to Health portal. Participants in the incentive group were eligible for weekly winnings based on weighing at least 6 days each week and received weekly feedback on their winnings to keep self-weighing goals salient. From months 7 to 12 (phase 2), participants received no further weekly messages or incentives tied to self-weighing.

Participants in the incentive group were informed of the structure of the escalating lottery at the onset of the intervention. Incentive group participants were eligible to win lotteries worth an expected value of $3.98 in week 1 that increased by $0.43 per week for each week that they achieved the goal of weighing 6 of 7 days. The lottery provides infrequent large payoffs (a 1-in-100 chance at a $110 reward in week 1) and frequent small payoffs (an 18-in-100 chance at a $16 reward in week 1) given that individuals are motivated by the future (fixation on large potential winnings) and the past (how often did I win?).^8,9^ Lotteries also have a theoretical advantage over direct payments in providing variable reinforcement, which has been demonstrated as more effective in reinforcing behavior than consistent reinforcement.^10^ The lottery was designed with rewards that start small and then escalate over time (as internal motivation begins to wane) and as weight maintenance becomes more difficult. Increasing the reward magnitude, conditional on ongoing engagement, is a way to leverage the endowment effect and loss aversion because participants face an ever-greater lost opportunity if they discontinue engagement.^11^ If participants missed the target 2 weeks in a row, they returned to the beginning of the escalating rewards schedule and were warned of this outcome the preceding week. If participants missed the target one week but adhered the next week, their potential reward would resume where they left off. When a participant won the lottery but did not meet the self-weighing goal, the participant was notified that he or she would have won because anticipated regret could motivate future adherence. Other than the total number of lottery weeks (24 weeks), there was no limit to the number of times a person could win the lottery. For participants who achieved all their weekly goals for the entire 24 weeks of the intervention, the expected value of the lottery, based on probabilities of winning the small and large lotteries, was approximately $214.

All participants were asked to complete online surveys at months 6 and 12 and received automated emails or text messages from the Way to Health platform notifying them of their compensation on completion. To enhance retention, participants received $50 each time for completing the online survey at the end of 6 and 12 months, independent of self-weighing adherence. All payments were approved by staff electronically from the Way to Health platform, and a text message was sent to participants alerting them of the payment. Payment information was transmitted to a bank to process and sent to participants via check weekly.

### Measurements

Weight was measured by each participant using the wireless scale. Physical activity was measured using the short form of the International Physical Activity Questionnaire,^12^ and eating habits were assessed with the Three-Factor Eating Questionnaire–R18, designed to assess three cognitive and behavioral domains of eating in obese individuals: cognitive restraint, uncontrolled eating, and emotional eating.^13,14^

### Safety Monitoring

The study was monitored by an independent data and safety monitoring board. Daily self-weighing data were also used to screen for unsafe weight loss strategies. Study staff contacted participants who lost more than 4.5 kg in 1 week or more than 9.0 kg in 1 month and asked about potential reasons for such weight loss, including unsafe strategies.

### Statistical Analysis

Participants were randomized in a 1:1 ratio stratified by sex and amount of weight lost (<13.6 vs ≥13.6 kg) in their first 4 to 6 months of WW using permuted block randomization with variable block sizes. The study was powered to detect between-group differences in weight change from baseline to the end of phase 1, when the intervention should achieve its maximal effect. Assuming a 5.0-kg SD for change in weight at 6 months based on a previous study^4^ and 20% missingness, there was 90% power to detect at least 2.3 kg more weight loss during 6 months for the intervention group compared with a control group.

Data were analyzed from May 23, 2016, through November 13, 2017. The primary analysis was an intention-to-treat, unadjusted, between-group comparison of the mean change in weight from baseline (randomization) to 6 months with 95% CIs, using an unequal-variance *t* test performed at the significance level of *P* < .05 and multiple imputation for handling of missing data. The 6-month weight was determined as the first self-weighing measurement that occurred inside a 4-week window starting at week 24. Our multiple imputation strategy used the following baseline covariates to impute missing data for the primary outcome: study group, baseline weight, age, sex, self-reported race, body mass index, educational level, annual income, qualifying weight loss, minutes of moderate and vigorous activity and walking from the International Physical Activity Questionnaire, the 3 domains of eating behaviors (cognitive restraint, uncontrolled eating, and emotional eating), and 2 scores related to delayed gratification behavior. We also estimated the difference between groups at 6 months in an adjusted analysis in which the change from baseline was regressed in a linear model on study group and the same baseline covariates as in the imputation model. Analyses of the secondary outcome, weight change from baseline to 12 months (phase 2), were conducted in a similar manner as the primary outcome.

Additional secondary analyses included a between-group comparison of the percentage of individuals who maintained their weight (defined as gaining ≤1.36 kg) at 6 and 12 months using a χ^2^ test. The percentage of weeks that individuals weighed in at least 6 times was also compared between groups for each phase using an unequal-variance *t* test. The association between the mean self-weighing frequency and amount of weight lost at the end of the phase was estimated using the Spearman correlation coefficient in a cross-sectional analysis for each phase. The trend over time for the frequency of self-weighing measurements was also compared between groups using a generalized estimating equation with an autoregressive working correlation model; specifically, the mean number of days per week was modeled and compared between study groups after adjusting for study week and a week-by-group interaction. Comparisons were also made of the changes in self-reported physical activity and the 3 domains of eating behaviors (cognitive restraint, uncontrolled eating, and emotional eating) at 6 and 12 months after initial weight loss, in analyses similar to those described above for the change in weight.

All reported hypothesis tests were 2 sided. *P* < .05 was considered statistically significant. Analyses were conducted using SAS, version 9.4 (SAS Institute Inc).

## Results

### Recruitment and Enrollment

**Figure 2.**
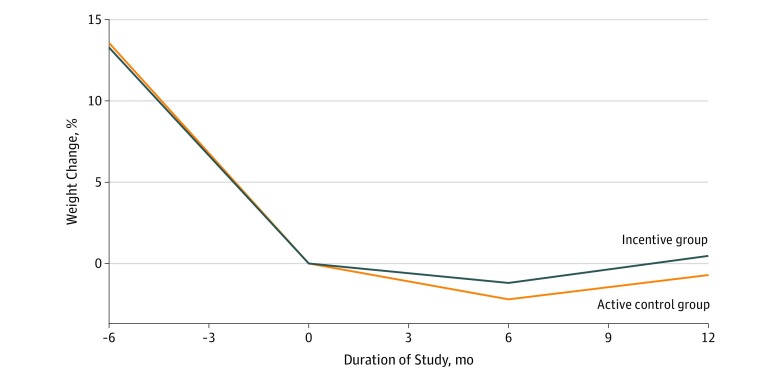
Mean Percentage Weight Change by Group From Entry Into Weight Management Program Over Time Month 0 indicates randomization. Mean weight changes were not different between the incentive (n = 128) vs active control (n = 130) groups at month 6 (mean difference, 0.7 kg; 95% CI, −0.7 to 2.2 kg; unequal-variance *t* test, *P* = .30) or month 12 (mean difference, 0.8 kg; 95% CI, −1.2 to 2.8 kg; unequal-variance *t* test, *P* = .41). *P* values were based on all participants, with multiple imputation to fill in the missing data at 6 months (n = 8) and 12 months (n = 31).

A total of 4847 WW Digital members received an invitation email; of these, 421 initiated the enrollment process, and 259 were randomized (Figure 1). After 1 patient in the incentive group was excluded becausue of being diagnosed with cancer, a total of 258 participants were included in the analysis (128 in the incentive group and 130 in the control group). The mean (SD) age of the participants was 48.0 (10.5) years; 225 (87.2%) were women and 33 (12.8%) were men; 235 (91.1%) were white; 149 (57.8%) had at least a college degree; and 102 (39.5%) had an annual income of at least $100 000 (Table 1). The mean (SD) weight loss before study enrollment was 11.6 (4.2) kg or 11.5% (3.7%) of original body weight (Figure 2); the mean (SD) body mass index at randomization was 32.1 (3.9).

**Table 1.** Baseline Participant Characteristics by Group

Variable	Study Group, No (%)^a^		
	All (N = 258)	Incentive (n = 128)	Active Control (n = 130)
Age, mean (SD), y	48.0 (10.5)	46.9 (10.2)	49.2 (10.6)
Sex			
Male	33 (12.8)	16 (12.5)	17 (13.1)
Female	225 (87.2)	112 (87.5)	113 (86.9)
Self-reported race			
White	235 (91.1)	115 (89.8)	120 (92.3)
Black	12 (4.7)	8 (6.3)	4 (3.1)
Other	11 (4.3)	5 (3.9)	6 (4.6)
Self-reported ethnicity			
Hispanic or Latino	15 (5.8)	6 (4.7)	9 (6.9)
Non-Hispanic or non-Latino	243 (94.2)	122 (95.3)	121 (93.1)
Educational level			
High school or less	33 (12.8)	11 (8.6)	22 (16.9)
Some college	76 (29.5)	34 (26.6)	42 (32.3)
College degree	74 (28.7)	41 (32.0)	33 (25.4)
Postgraduate	75 (29.1)	42 (32.8)	33 (25.4)
Annual income			
<$50 000	29 (11.2)	14 (10.9)	15 (11.5)
$50 000-$99 999	127 (49.2)	65 (50.8)	62 (47.7)
$100 000-$149 999	76 (29.5)	35 (27.3)	41 (31.5)
≥$150 000	26 (10.1)	14 (10.9)	12 (9.2)
Willing to give up something today to benefit in the future			
Most averse to risks	9 (3.5)	3 (2.3)	6 (4.6)
Moderately averse to risks	183 (70.9)	87 (68.0)	96 (73.8)
Least averse to risks	66 (25.6)	38 (29.7)	28 (21.5)
Postpone things although it would be better to get done right away			
Most averse to risks	89 (34.5)	45 (35.2)	44 (33.8)
Moderately averse to risks	135 (52.3)	65 (50.8)	70 (53.8)
Least averse to risks	34 (13.2)	18 (14.1)	16 (12.3)
Baseline IPAQ level			
Low	48 (18.6)	28 (21.9)	20 (15.4)
Moderate	107 (41.5)	55 (43.0)	52 (40.0)
High	102 (39.5)	44 (34.4)	58 (44.6)
Missing^b^	1 (0.4)	1 (0.8)	0
Household size, mean (SD), No. of persons	3.0 (1.4)	3.2 (1.6)	2.8 (1.3)
BMI at randomization, mean (SD)	32.1 (3.9)	32.6 (4.2)	31.7 (3.7)
Weight loss before randomization, mean (SD), kg	11.6 (4.2)	11.8 (4.1)	11.5 (4.3)
Activity by IPAQ, median (IQR), min/wk^b^^,^^c^			
Moderate to vigorous	150.0 (40.0-305.0)	145.0 (50.0-270.0)	180.0 (30.0-390.0)
Walking	210.0 (100.0-420.0)	210.0 (105.0-420.0)	210.0 (100.0-480.0)
Total MET	1853.0 (982.5-3582.0)	1653.0 (982.5-2940.0)	2044.5 (918.0-4158.0)
Domain of eating behavior, mean (SD) score^d^			
Cognitive restraint	63.7 (14.3)	64.1 (15.6)	63.2 (12.9)
Uncontrolled eating	42.2 (17.9)	42.7 (17.9)	41.8 (17.9)
Emotional eating	52.6 (24.9)	53.9 (24.5)	51.4 (25.4)

Abbreviations: BMI, body mass index (calculated as weight in kilograms divided by height in meters squared); IPAQ, International Physical Activity Questionnaire; IQR, interquartile range; MET, metabolic equivalent of task.

^a^ Percentages have been rounded and may not total 100.

^b^ One participant from the incentive group had physical activity data at baseline excluded because it was considered an outlier by the IPAQ scoring protocol.

^c^ Measured at baseline.

^d^ Measured at baseline using the Three-Factor Eating Questionnaire–R18. Scores range from 18 to 76, with higher scores indicating higher cognitive restraint and emotional and uncontrolled eating.

### Primary Weight Outcome

Participants in both groups successfully maintained their initial weight loss during the 6 months of the intervention (phase 1) and the additional 6 months of follow-up after the intervention (phase 2) (Table 2 and Figure 2). The 6-month weight outcome was observed in 250 (96.9%) of the 258 participants in the analysis cohort; the 12-month weight outcome, in 227 (88.0%). In the intention-to-treat analysis, mean weight changes at the end of phase 1 were −1.1 (95% CI, −2.1 to −0.1) kg in the incentive group and −1.9 (95% CI, −2.9 to −0.8) kg in the control group, with a mean difference of 0.7 (95% CI, −0.7 to 2.2) kg (*P* = .30 for comparison). Mean weight changes from baseline to the end of phase 2 (end of study) were 0.2 (95% CI, −1.2 to 1.7) kg in the incentive group and −0.6 (95% CI, −2.0 to 0.8) kg in the control group, with a mean difference of 0.8 (95% CI, −1.2 to 2.8) kg (*P* = .41 for comparison). Analyses adjusted for baseline covariates yielded similar results, with a mean difference between groups in weight change from baseline to the end of phase 1 of 1.2 (95% CI, −0.2 to 2.5) kg (*P* = .09 for comparison) and from baseline to the end of phase 2 of 1.2 (95% CI, −0.8 to 3.2) kg (*P* = .23 for comparison). Using a complete case analysis, results were similar to the intention-to-treat analysis; mean weight changes from baseline to the end of phase 1 were −1.1 (95% CI, −2.2 to −0.1) kg in the incentive group and −1.8 (95% CI, −2.8 to −0.8) kg in the control group; from baseline to end of phase 2, 0.4 (95% CI, −1.0 to 1.8) kg in the incentive group and −0.4 (95% CI, −1.9 to 1.1) kg in the control group. The percentage of incentive vs control participants who maintained their weight (defined as gaining ≤1.36 kg) at 6 months was 79.2% (95% CI, 72.0%-86.4%) vs 82.2% (95% CI, 75.3%-89.0%) (*P* = .55 for comparison); at 12 months, 65.4% (95% CI, 56.4%-74.4%) vs 67.8% (95% CI, 59.3%-76.4%) (*P* = .67).

**Table 2.** Mean Changes in Weight, Physical Activity, Eating Habits, and Self-weighing Frequency by Group

Change Variable	Effect (95% CI)		Incentive vs Control Groups	
	Incentive Group (n = 128)	Active Control Group (n = 130)	Effect (95% CI)	*P* Value^**a**^
Mean weight, kg				
6 mo	−1.1 (−2.1 to −0.1)	−1.9 (−2.9 to −0.8)	0.7 (−0.7 to 2.2)	.30
12 mo	0.2 (−1.2 to 1.7)	−0.6 (−2.0 to 0.8)	0.8 (−1.2 to 2.8)	.41
Mean total activity, MET, min/wk				
6 mo	−63.4 (−398.8 to 272.0)	−323.7 (−684.7 to 37.3)	260.3 (−230.0 to 750.6)	.30
12 mo	141.0 (−236.6 to 518.5)	197.5 (−156.5 to 551.6)	−56.5 (−571.3 to 458.2)	.83
Mean MVPA activity, min/wk				
6 mo	−3.4 (−49.3 to 42.5)	−39.6 (−91.8 to 12.6)	36.2 (−32.9 to 105.3)	.30
12 mo	23.4 (−20.4 to 67.2)	77.1 (26.5 to 127.7)	−53.7 (−120.3 to 12.9)	.11
Mean walking, min/wk				
6 mo	−6.0 (−74.7 to 62.7)	−30.5 (−100.4 to 39.4)	24.5 (−73.0 to 122.0)	.62
12 mo	141.0 (−236.6 to 518.5)	197.5 (−156.5 to 551.6)	−56.5 (−571.3 to 458.2)	.83
Mean Cognitive Restraint Scale score^b^				
6 mo	−2.9 (−5.5 to −0.4)	−3.2 (−5.8 to −0.6)	0.3 (−3.4 to 3.9)	.89
12 mo	−8.7 (−12.3 to −5.0)	−4.5 (−7.3 to −1.7)	−4.1 (−8.7 to 0.4)	.08
Mean Uncontrolled Eating Scale score^b^				
6 mo	−1.2 (−3.7 to 1.3)	1.0 (−1.1 to 3.2)	−2.2 (−5.5 to 1.0)	.17
12 mo	0.4 (−2.9 to 3.7)	0.3 (−2.2 to 2.8)	0.1 (−4.0 to 4.2)	.97
Mean Emotional Eating Scale score^b^				
6 mo	−0.7 (−4.5 to 3.2)	0.8 (−2.2 to 3.8)	−1.5 (−6.4 to 3.4)	.56
12 mo	0.2 (−4.5 to 4.9)	−1.1 (−4.7 to 2.4)	1.3 (−4.5 to 7.2)	.65
Maintained weight, %^c^				
Baseline to 6 mo	79.2 (72.0 to 86.4)	82.2 (75.3 to 89.0)	−3.0 (−12.9 to 6.9)	.55
Baseline to 12 mo	65.4 (56.4 to 74.4)	67.8 (59.3 to 76.4)	−2.4 (−14.8 to 9.9)	.67
6 to 12 mo	58.2 (48.9 to 67.5)	62.2 (53.3 to 71.1)	−4.0 (−16.8 to 8.8)	.53
Mean at-home weigh-in frequency, times/wk				
Phase 1	6.2 (6.0 to 6.4)	5.8 (5.6 to 6.1)	0.4 (0.1 to 0.7)	.02
Phase 2	3.6 (3.2 to 4.0)	4.4 (4.0 to 4.8)	−0.8 (−1.3 to −0.2)	.009

Abbreviations: IPAQ, International Physical Activity Questionnaire; MET, metabolic equivalent of task; MVPA, moderate to vigorous physical activity.

^a^ Comparisons are calculated based on all participants, with multiple imputations to address missing data when necessary. Six-month weight was missing for 8 participants; 12-month weight for 31; 6-month IPAQ scores for 17; 12-month IPAQ scores for 37; 6-month Three-Factor Eating Questionnaire–R18 scores for 16; and 12-month Three-Factor Eating Questionnaire–R18 scores for 37. Unless otherwise indicated, comparisons between groups are calculated using unequal-variance *t* tests.

^b^ Calculated using the Three-Factor Eating Questionnaire–R18. Scores range from 18 to 76, with higher scores indicating higher cognitive restraint and emotional and uncontrolled eating.

^c^ Comparison between groups are calculated using χ^2^ tests.

### Secondary Outcomes

**Figure 3.**
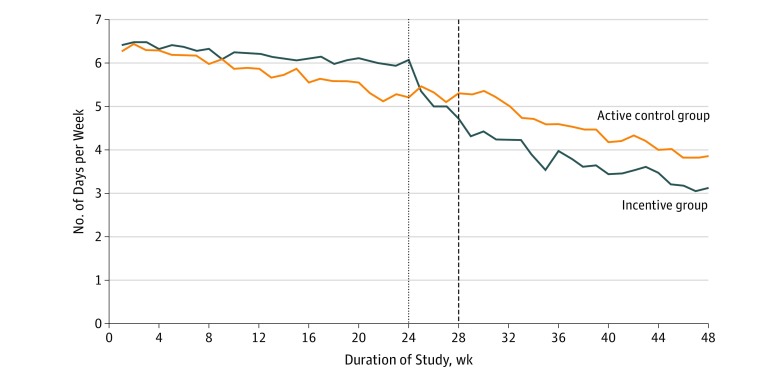
Mean Self-weighing Frequency Self-weighing frequency was measured as mean number of days per week that self-weighing was performed by group over time. Week 0 indicates randomization. Self-weighing frequency in the incentive group (n = 128) declined more slowly than in the active control group (n = 130) during weeks 0 to 24 (difference, 0.03 times/wk; 95% CI, 0.01-0.05 times/wk; Wald test, *P* = .002) with similar declines in the 2 groups during weeks 28 to 48 after a transition period during which incentives ceased in the incentive group.

During the 6-month intervention (phase 1), the percentage of weeks that incentive and control participants achieved mean self-weighing at least 6 times was 85.3% vs 75.8%, respectively (*P* = .002 for comparison). After an additional 6 months of follow-up without intervention (phase 2), the percentages were 37.7% vs 50.2% for the intervention and control groups, respectively (*P* = .009). The trends in self-weighing frequency over time by group (Figure 3) demonstrated a decrease in frequency immediately after cessation of incentives in that group. The mean self-weighing frequency declined for both groups over time, with the incentive group having a slower decline in phase 1 (difference, 0.03 [95% CI, 0.01-0.05] times per week; Wald test *P* = .002 for comparison) and similar declines in phase 2 after the transition period (difference, 0.02 [95% CI, −0.01 to 0.04] times per week; *P* = .26). Across both study groups, self-weighing frequency was associated with weight change in phase 1 (−0.34 [95% CI, −0.45 to −0.22] times per week) and phase 2 (−0.25 [95% CI, −0.38 to −0.11] times per week). In other words, the greater the weighing frequency, the smaller the gains (or larger the losses) were for weight. At 6 and 12 months after initial weight loss, changes in self-reported minutes of moderate or vigorous physical activity and walking and changes in the 3 domains of eating behaviors (cognitive restraint, uncontrolled eating, and emotional eating) were not statistically significantly different across groups (Table 2).

During the phase 1 weight loss maintenance intervention, 365 incentive payments (total of $17 600) were made to the incentive participants (mean [SD] per participant, $137.50 [$157.82]; maximum, $875.00; minimum, $0.00). Incentive group participants adhered to the self-weighing requirement in consecutive weeks well enough to step up their reward a mean of 11 (of a possible 23) times before their first nonadherent week, which would translate into $27 in escalated small lottery winning or $385 in escalated large lottery winning. Among incentive group participants, 33 (25.8%) were adherent every week in phase 1, and 76 (80.9%) were adherent the week after a nonadherent week. In contrast, 35 (27.3%) did not self-weigh 6 of 7 days for 2 weeks in a row, returning them to the baseline incentive amount at least once during phase 1.

### Excess Weight Loss Events and Other Adverse Events

A total of 71 weight loss alerts, including alerts for 66 participants with a loss of at least 4.5 kg in 1 week and 5 with a loss of at least 9 kg in 1 month, occurred during 12 months of follow-up. No unhealthy weight loss behaviors were reported. The most common reason for an alert was another family member using the scale (15 of 71 alerts [21.1%]) followed by scale calibration error (14 [19.7%]), resumption of diet or exercise (9 [12.7%]), illness (8 [11.3%]), and abnormal time or apparel (8 [11.3%]). Fourteen serious adverse events were reported by participants throughout the study, none of which were related to the study.

## Discussion

In this study, we found no evidence that escalating lottery rewards resulted in significantly greater maintenance of weight loss than in an active control group. This finding occurred despite the incentives successfully increasing the frequency of participants weighing in at least 6 times per week. The incentive and control groups successfully maintained their initial weight loss, highlighting that this trial was conducted within a population of self-selected WW Digital members who not only achieved significant initial weight loss but who also were a small percentage (5.3%) of those eligible who chose to participate in this study.

Earlier work^2,3,4^ indicated that a greater frequency of self-weighing was associated with greater weight loss. Based on that observation, we tested the premise that increasing the proportion of participants who self-weighed 6 or 7 days per week would enhance weight maintenance or increase further weight loss. Although we observed a 20–percentage point difference in the proportion of participants self-weighing at least 6 days per week, we did not observe any appreciable difference in maintenance of weight loss. Each of the following may have contributed to this finding: (1) in this self-selected group, participants in both groups were intrinsically motivated and did not need further inducements to succeed in maintaining weight loss; (2) the active control condition (with weekly feedback and wireless scale) was sufficiently potent that financial incentives provided no enhancement; (3) the incentive amounts or design were not sufficiently motivating; (4) the observation from the previous study^4^ reflected differences in the type of people who self-weigh frequently rather than a causal relationship with weight loss; or (5) the degree of difference in self-weighing frequency was insufficient to lead to a difference in weight loss.

Previous research on incentives related to obesity^15,16,17,18^ focused on initial weight loss and found incentives to be relatively effective, but we are aware of only 1 previous study on incentives for maintenance of weight loss.^4^ In a similar population of WW members who had achieved initial weight loss, that study similarly found that 2 types of incentives (direct incentive and lottery incentive aimed at weight outcomes) were no more successful than an active control in maintaining weight loss, but all groups were successful in maintaining their weight loss.^4^

One especially interesting finding is that, although the incentive participants self-weighed more effectively than the control participants in phase 1 when the incentives were in effect, they self-weighed less frequently during phase 2 after the incentives were removed. This effect could potentially be viewed as a “crowding out” of intrinsic motivation during phase 2 in the incentive group. This outcome is an important theoretical concern but did not result in a differential effect on weight in either phase.

### Limitations

This work had some limitations. This study and the previous study^4^ among people who had successfully achieved initial weight loss in an existing, non–research-based weight management program highlight the difficulty of enrolling such a population into a maintenance intervention because only 5.3% of the eligible population chose to participate, and that 5.3% may have been more highly motivated than the rest of the population from which we recruited as well as the general population. This issue is important in consideration of the design and generalizability of such studies. Typically, real-world efficacy testing is considered a preliminary step to testing population-level effectiveness.^19^ However, in these cases, the self-selected control group of volunteers who agreed to participate likely did better than the rest of the eligible population, and the fact that both study groups successfully maintained weight loss made it more challenging for the intervention to demonstrate superior efficacy. This difficulty suggests that, for interventions that may have a natural ceiling effect associated with maintenance of existing goals, testing effectiveness in a less selected population may provide more useful insights in terms of potential effect on population health. In addition to the select sample, another limitation is that we enrolled participants from only 1 commercial weight management program; although it is the largest commercial weight loss program in the world, the participants in the WW Digital program may have unique characteristics compared with others who have overweight and obesity in the United States, further limiting generalizability of the study findings.

## Conclusions

In summary, we found that escalating incentives meant to encourage more frequent self-weighing in a population of volunteers who had experienced significant initial weight loss in a commercial weight management program succeeded in increasing the proportion of participants who weighed themselves at least 6 times per week but did not succeed in improving weight loss maintenance. However, both groups were successful in maintenance of initial weight loss, suggesting that, in this self-selected group of volunteers, further incentives, in addition to a wireless scale and weekly feedback, were not necessary to help them achieve weight loss maintenance.

## Notes

Supplement 1. **Trial Protocol**

Supplement 2. **Data Sharing Statement**
