# Supplementary material for: Effect of Escalating Financial Incentive Rewards on Maintenance of Weight Loss: A Randomized Clinical Trial
Source: JAMA Netw Open. 2019 Nov 1;2(11):e1914393. doi: 10.1001/jamanetworkopen.2019.14393 (PMC6826643; doi:10.1001/jamanetworkopen.2019.14393)
Supplement: Supplement 1. — Trial Protocol [file jamanetwopen-2-e1914393-s001.pdf]

Escalating financial incentive rewards for maintenance of weight loss: an internet-based randomized controlled trial

Trial Registration: clinicaltrials.gov Identifier NCT01900392; https://clinicaltrials.gov/ct2/show/NCT01900392

William S. Yancy Jr., MD, MHSc[1,2,3]; Pamela A. Shaw, PhD[4,5]; Catherine Reale, BS[5,6,7]; Victoria Hilbert, MPH, RD[5,8]; Jiali Yan, MS[9]; Jingsan Zhu, MS, MBA[5,8]; Andrea B. Troxel, ScD[10]; Gary D. Foster, PhD[11,12,13]; Kevin G. Volpp, MD, PhD[5,6,8,9,14]

January 24, 2018; Version 5

Funding: This study is funded through National Institute On Aging of the National Institutes of Health, Award Number R01-AG045045 (Volpp and Yancy, MPIs).

1. Duke University Diet and Fitness Center, Durham, NC

2. Department of Medicine, Duke University School of Medicine, Durham, NC

3. Center of Innovation to Accelerate Discovery and Practice Transformation (ADAPT), Department of Veterans Affairs, Durham, NC

4. Department of Biostatistics, Epidemiology and Informatics, Perelman School of Medicine, University of Pennsylvania, Philadelphia, PA

5. Leonard Davis Institute Center for Health Incentives and Behavioral Economics, University of Pennsylvania Perelman School of Medicine, Philadelphia, PA

6. Department of Health Care Management, The Wharton School, University of Pennsylvania, Philadelphia, PA

7. Center for Clinical Epidemiology and Biostatistics, Perelman School of Medicine, University of Pennsylvania, Philadelphia, PA

8. Department of Medical Ethics and Health Policy, Perelman School of Medicine, University of Pennsylvania, Philadelphia, PA

9. Department of Medicine, Perelman School of Medicine, University of Pennsylvania, Philadelphia, PA

10. Department of Population Health, New York University School of Medicine, New York, NY
11. Department of Science, WW (formerly Weight Watchers), New York, NY
12. Center for Obesity Research and Education, Temple University, Philadelphia, PA
13. Center for Weight and Eating Disorders, Perelman School of Medicine, University of Pennsylvania, Philadelphia, PA
14. Center for Health Equity Research & Promotion, Philadelphia Veterans Affairs Medical Center, Philadelphia, PA

Study Instruments

The proposed study will evaluate the effectiveness of financial incentives in improving maintenance of weight loss. We propose a 2-phase, 2-arm randomized controlled trial (RCT) in which 259 participants who lose at least 5 kilograms (11 pounds) in a weight loss program will be randomized to receive one of the following: 1) daily weigh-ins and weekly feedback (control arm) or 2) daily weigh-ins, weekly feedback, and a weekly escalating lottery-based financial incentive (Phase I, months 1-6). Those assigned to the escalating lottery condition will be eligible to win lotteries worth an expected value of $3.98 in week 1 that will increase by $0.43 per week for each week that they achieve the goal of weighing 6/7 days. Participants in all arms will be observed without intervention in Phase II.

Method for Assigning Subjects to Groups

Potential participants will be identified by reviewing Weight Watchers online member records using our eligibility criteria of initial BMI upon entry into the program, pounds lost in the last 4-6 months, and member opted in for communications from Weight Watchers about research studies. Weight Watchers will facilitate communication to these members using existing communication channels (email). Potentially eligible individuals will be invited to visit the Way to Health portal, an NIH-funded web-based infrastructure based at the University of Pennsylvania that is used to run behavioral economic intervention studies. Participants meeting the inclusion criteria will be randomized to one of the 2 study arms through the Way to Health platform. We will use permuted block randomization with variable block sizes. Participants are randomized in a 1:1 ratio to the

intervention or control arm and randomization will be stratified by: (1) sex and (2) amount of weight lost within first 4-6 months of Weight Watchers membership (<30 lbs. versus 30+ lbs), as baseline weight loss may moderate treatment effects. Prior to a participant signing the informed consent, both the participant and the study coordinator will be blinded to what their potential group assignment of the participant would be if enrolled. Once consented and enrolled, neither the participants nor the study coordinator will be blinded to condition assignment due to the nature of the interventions. However, the consent form will not elucidate the design of the two intervention arms so that participants are not aware of the comparisons being made. For scientific purposes to establish valid measures of the effects of the various kinds of incentive plans, blinding participants to the overall study design is necessary, presents no increment of risk, and doesn't violate subject rights. Once a subject has been assigned to a study arm, she will receive an automatically-generated email notification about group assignment and next steps. At the close of the study, all participants will receive a debriefing letter disclosing all study arms and informing participants of the final results of the study (using de-identified data) that will be submitted for publication to an academic journal at the completion of the analysis.

Administration of Surveys and/or Process

Basic demographic data (BMI, gender, race, education, income, household size), physical activity data (IPAQ long), and eating behavior questions (TFEQ) and 2 questions measuring delayed gratification will be collected upon enrollment. We will also collect this data, minus the demographic questions, at 6 and 12 months.

Protocol

Abstract

Obesity is the second leading cause of preventable death in the United States, (1) associated with high blood pressure (BP), type 2 diabetes, coronary heart disease, and osteoarthritis. Identifying effective strategies for treating obesity is both a clinical challenge and a public health priority. Over 70% of the U.S. population is overweight or obese, and obesity in middle age translates into higher rates of hospitalization, Medicare expenditures, disability, and mortality risk in subsequent years. While a variety of approaches are successful in achieving initial weight loss,

techniques for maintenance of initial weight loss have largely been unsuccessful.

Our previous work examining the impact of different degrees of cost sharing on employee participation, engagement, and ongoing weight loss showed that among Weight Watchers members who had initial success in losing at least 5% of their initial body weight, the majority regained weight over the subsequent 6 months; Weight Watchers members in general were at high risk of dropping out of the program altogether perhaps due to lack of success in losing weight and keeping it off (John et al 2015). Research has shown that the frequency of self-weighing is important for both prevention of weight gain and weight loss (Wing et al NEJM 2006; 355:1563-1571; Linde JA et al Ann Behav Med 2005; 30: 210-216). This study will build on our successful efforts using incentives for weight loss to test scalable ways of increasing the frequency of self-weighing among Weight Watchers members who had initial success in losing weight. This 2- arm randomized controlled trial will compare the relative effectiveness and cost-effectiveness of (1) weekly feedback without incentives to (2) weekly escalating lottery-based incentives among patients who successfully lost at least 5 kg over 4-6 months during participation in Weight Watchers (Pre Phase). Incentives will be provided to some study participants (Arm 2) for the first 6 months (Phase I), and then participants will be followed for 6 more months to examine effects following cessation of incentives (Phase II). A total of 259 participants will be randomized into the 2 arms.

Specific Aims

Aim 1: Among participants who lose 5 kg or more of baseline weight prior to randomization, assess the effectiveness of escalating lottery rewards, relative to the control group, on maintenance of weight loss over the ensuing 6 months (Phase I).

Aim 2: Assess the degree to which weight loss is maintained in the intervention group relative to usual care during the 6 months following the cessation of the interventions (Phase II).

Objectives

Overall objectives

1. Among participants who lose 5 kg or more of baseline weight prior to randomization, assess the effectiveness of escalating lottery rewards, relative to the control group, on maintenance of weight loss.

2: Assess the degree to which weight loss is maintained in the intervention groups relative to usual care during the 6 months following the cessation of the interventions (Phase II).

Primary outcome variable

Pounds of weight lost at the end of 6 months

Secondary outcome variables

1. Pounds of weight lost at the end of 12 months
2. Percent of individuals who maintained their weight (defined as gaining no more than 1.36 kg or 3 lbs) at 6 and 12 months
3. Average weekly weigh-in frequency during Phase I
4. Average weekly weigh-in frequency during Phase II
5. Linear trend for weigh-in frequency during Phase I
6. Linear trend for weigh-in frequency during Phase II
7. Self-reported physical activity at 6 months, as measured by total MET-minutes/week, total minutes in Moderate to Vigorous Activity (MVPA), and minutes/week walking
8. Self-reported physical activity at 12 months, as measured by total MET-minutes/week, total minutes in Moderate to Vigorous Activity (MVPA), and minutes/week walking
9. Eating behavior at 6 months (cognitive restraint, uncontrolled eating, emotional eating)
10. Eating behavior at 12 months (cognitive restraint, uncontrolled eating, emotional eating)
11. Proportion of the time people fall off the lottery escalation ladder
12. Number of resets to beginning for the lottery winnings

Exploratory outcome variable

Frequency of weight self-reported to Weight Watchers online

Background

Obesity is the second leading cause of preventable death in the United States, associated with high blood pressure (BP), type 2 diabetes, coronary heart disease, and osteoarthritis. Weight loss of just 5-10 kg can improve risk factors (e.g., BP, glycemia, and serum lipid levels) and reduce the incidence of diabetes. In older adults, weight loss interventions have shown clear improvements in BP, arthritis, and functional status. However, maintenance of weight loss is needed to achieve long term health benefits, and successful strategies for long-term maintenance are lacking. Weight regain after a period of intentional weight loss is widely observed with all interventions due to loss of motivation, lack of sustained rewards for weight loss behavior, difficulty adhering to diet, and need for ego-depleting exertion of willpower. For maximum generalizability, we will enroll study participants from Weight Watchers [WW]), the largest commercial weight loss program nationally. Since this program, like other successful weight loss interventions, has difficulty with weight loss maintenance, an external motivational source such as financial incentives may help people keep weight off better than standard approaches. Individuals put disproportionate value on the present relative to future costs and benefits, known as present-biased preferences. While this bias typically works against healthy behavior, the same factors can be used to promote compliance by providing tangible but small immediate rewards for beneficial behaviors. The literature most relevant to obesity interventions relates to the impact of incentives on addiction-related behaviors, which, like diet, are difficult to change and involve a conflict between an individual's long-term well-being and short-term cravings. As reported in the Contingency Management literature, financial incentives have been highly effective for such patients, leading to higher rates of program retention and abstinence. However, the most effective approaches are those that require monitoring several times a week, suggesting the importance of frequent feedback. A review of 11 randomized trials of financial incentives found that financial incentives promoted adherence better than any tested alternative, leading to better blood pressure control, appointment attendance, and higher immunization rates. Other reviews have found various incentives (including lotteries) effective in changing behavior. However, few studies have examined cost-effectiveness or longer-term effects of incentives on health behaviors after cessation of incentives, or the relative effectiveness of traditional economic incentives versus behavioral economic incentives that consider the underlying psychology of decision making. The most compelling early work on incentives for weight loss was done by Dr. Robert Jeffery. He showed

that participants without any weight management training lost weight when they deposited valuables with a therapist with return of their valuables contingent on progress towards pre-specified goals, and that incentives for weight loss are more effective than incentives for attending weight loss training sessions. Finkelstein et al. found that over a 3-month period, participants offered $14 per percentage point of weight loss lost 4.7 pounds whereas participants offered $7 lost 3.0 pounds and control participants lost 2.0 pounds. Incentives for maintenance of weight loss ($10 for participants who gained weight) demonstrated higher rates of maintaining weight loss that were not statistically significant. These studies illustrate that incentives, even if small and infrequent, can be effective; higher rewards and more frequent feedback could substantially enhance these effects. Recent studies concluded that annual US expenditures attributable to overweight and obesity are between $90 and $100 billion. Among nationally representative cohorts, the average annual incremental cost of obesity per person is about $1308. Higher BMIs in young adulthood and middle age are associated with substantially higher Medicare expenditures in old age. Higher BMI also translates into significantly higher health care charges within 18 months, suggesting that health plans may find it cost effective to invest in behavior modification to lower health expenditures. Several economic assessments of behavioral interventions for weight loss have been published. The Diabetes Prevention Program, reported that, compared to placebo, the intensive lifestyle intervention had cost per QALY ratios ranging between $32,000 and $52,000, depending upon whether direct medical costs alone or direct and indirect costs were counted. A comparison of low carbohydrate and low fat diets by Glick et al suggested that low carbohydrate diets may be cost effective. Finkelstein et al. reported that the 1-year incremental cost per 1% reduction in 10-year CHD risk associated with an enhanced behavioral intervention was $637. The substantial health and economic consequences of obesity highlight the importance of testing new approaches to maintenance of weight loss and the potential cost effectiveness of successful interventions.

Study Design

Design

Study participants will be obese (at time of enrollment in Weight Watchers) volunteers recruited from a major

community-based weight loss program (Weight Watchers (WW)), who have lost at least 5 kg over their first 4-6 months in the program. Eligible participants will be randomized to a control group (weekly feedback via text and email messaging and regular measurement of weight for 6 months), or an escalating lottery-based financial incentive. Participants will be asked to weigh in on a digital scale 6/7 days each week. Participants in the incentive arm will be eligible for weekly winnings based on weighing 6/7 days each week and will receive weekly feedback on their winnings to keep weighing goals salient. Participants in both arms will receive weekly tips on how to make weighing in frequently easier to do. There will be some diet & exercise-related tips mixed in with weighing in frequently tips. Between months 7-12 (Phase II) participants will receive no further incentives tied to weighing, nor will they receive a weekly tip. The study is powered to test the effectiveness of the intervention arm relative to control.

Study duration

We estimate it will take 9 months to enroll 259 participants. The intervention will last for 6 months. We will remove the intervention and observe changes in weight over the subsequent 6 months for a total length of participation of 12 months.

Characteristics of the Study Population

Target population

Eligible participants will be men and women ages 30 to 80 years who had a BMI of 30 to 45 kg/m^2 prior to starting Weight Watchers, have a documented weight loss of at least 5 kg in the past 4-6 months, are in stable health, and have an online Weight Watchers membership.

Accrual

Weight Watchers has over 1 million members. Weight Watchers will send invitations to their online members that

meet our inclusion criteria and opted in for communications from Weight Watchers about research studies. Given the large pool of potential participants, we should be able to recruit 259 participants in 9 months.

Key inclusion criteria

(1) Individuals ages 30 to 80 years (2) BMI of 30 to 45 kg/m2 prior to starting Weight Watchers (3) Have a documented weight loss of at least 5 kg in the first 4-6 months of joining Weight Watchers; (4) In stable health; (5) Have an online Weight Watchers membership. The age range represents those ages most affected by obesity in terms of prevalence, and of associated disease, disability, and healthcare costs. Instead of targeting only older adults, we extend the lower age range to 30 years because obesity in middle age has a strong association with disability in later years. The upper limit of BMI will minimize the influence of outliers on the main result of weight loss and maximize the effect of incentives.

Key exclusion criteria

substance abuse; bulimia nervosa or related behaviors; pregnancy or breast feeding; medical contraindications to counseling about diet, physical activity, or weight reduction; unstable mental illness; screen positive for pathologic gambling on the basis of the 2 item Lie-Bet criteria (excluded if meets 1 or both criteria). Individuals unable to read consent forms or fill out surveys in English will be excluded.

Subject recruitment

Potential participants will be identified by Weight Watchers using our inclusion criteria. Weight Watchers will facilitate communication to these members using existing communication channels (email). Potentially eligible individuals will be invited to visit the Way to Health portal, an NIH-funded web-based infrastructure based at the University of Pennsylvania that is used to run behavioral economic intervention studies. Interested participants will be directed to the Way to Health platform where they will provide informed consent. These individuals will also be asked to enter data related to eligibility and their demographic and clinical characteristics through the Way to Health internet portal.

To enhance retention, we will provide a total of $100 to participants in all arms. Participant compensation will be independent of degree of weight loss and randomization group as follows: $50 for completing an online survey at the end of month 6 and at the end of month 12. All participants will also receive a free scale (value $179). All payments will be approved electronically from the Way to Health platform. Payment information will be sent to Wells Fargo to process and sent to participants via check. Participants will receive an automated message from the Way to health platform notifying them of compensation after completing each required survey. Participants in the financial incentive arm of the study will receive additional payments based on their adherence to daily weighing at home as outlined below in Study Procedures.

Statistical Methods

Sample size justification

We have designed the study with adequate power to detect differences in maintenance of initial weight loss. Our intervention should achieve its maximal impact in maintaining initial weight loss at the end of Phase I, when the incentive payments cease. The primary analysis will be an intent-to-treat analysis that compares the weight loss at 6 months between the lottery and control arms, using a t-test at the 0.05 level for significance. Assuming 20% missingness, a 5 kg (11 lb) SD for weight change at 6 months, and at least 5 lb more weight loss for the intervention arm compared to control at 6 months, we will have at least 90% power to detect the improvement in our primary outcome.

Analytic approach

Primary and key secondary outcomes

The primary analysis will be an unadjusted between-arm comparison of the change in weight from baseline

(randomization) to 6 months, using a t- test performed at the 0.05 significance level. The primary analysis will be done as an intent-to-treat (ITT) analysis, including each participant in the group to which she was randomized, regardless of adherence to the assigned intervention. The 6-month weight is determined as the first weight that is inside the 6-month visit window. A multiple imputation strategy based on baseline covariates (including study arm) will be used to fill in the missing data for the primary outcome. For more details see multiple imputation strategy below. A complete case analysis will be done as a sensitivity analysis. A second sensitivity, assuming weight returned to baseline (weight at time of randomization) may also be considered. We will also estimate the difference between arms at 6-month in an adjusted analysis where the change from baseline is regressed in a linear model on study arm, baseline weight, and other covariates including age, sex, race, BMI, education, income, and physical activity (total MET-minutes/week).

Analyses of the key secondary outcome, weight change between baseline and 12 months (Phase 2) will be conducted in a similar manner as the primary outcome. The 12-month weight is determined as the weight that is the first weight inside the 12-month visit window. The multiple imputation algorithm for the 12-month outcome will additionally consider the 6-month weight outcome as an additional predictor, using a chained imputation type approach.

Multiple Imputation Strategy

Missing primary outcome data, weight at 6 months, will be imputed using baseline covariates only (Imputation A); variables include age, sex, race, weight, BMI qualifying weight loss, education, income, IPAQ activity level, MET-minutes walking, the three domains of eating behaviors (cognitive restraint, uncontrolled eating, emotional eating, and two scores related to delayed gratification behavior. A supportive analysis will also be done also using post-randomization variables (Imputation B); these variables include the most recent logged weight and time since last weight, The imputation strategy for the weight at 12 months and other secondary outcomes will proceed in a similar manner. Weight at 12 months will additionally use the 6-month weight outcome, using chained imputation approach (1).

Analysis of other outcomes

The trend over time for the frequency of at-home weight measurements will be compared between arms using a generalized estimating equation (GEE) with an autoregressive (AR-1) working correlation model; specifically the mean number of days out of 7 is modeled and compared among study arms adjusting for study week and a week-by-arm interaction. Separate models will be fit for Phases 1 and 2. A linear mixed effects model with random intercept and slope for week will also be considered. An overall average weekly frequency of weighing in will be compared between arms for Phase I and Phase II using a similar GEE approach.

Additional secondary analyses include a between-arm comparison of the percent of individuals who maintained their weight (defined as gaining no more than 1.36 kg or 3 lbs) at 6 and 12 months. We will consider whether the amount of weight gain is correlated with the average number of times per week participants weighed themselves in a cross-sectional analysis at 6 and 12 months. Comparisons will also be made of the changes in self-reported physical activity and the three domains of eating behaviors (cognitive restraint, uncontrolled eating, emotional eating) at 6 months and 12 months after initial weight loss. Descriptive analyses will be done to examine the proportion of the time people fall off the lottery escalation ladder and the number of resets to beginning for the lottery incentive arm. All secondary analyses will follow in a manner similar to the primary analysis, considering both an unadjusted and adjusted approach. Imputation models for the secondary outcomes will include as predictors the baseline value for that outcome and the other baseline covariates considered for the imputation strategy A model, as described above. For secondary outcomes with < 10% data missing data, only a single imputation strategy (imputation A) may be considered.

Exploratory Analyses

We will summarize the frequency of the self-reported weights in Phase I and Phase II and examine whether there will be a difference by arm. We will further explore intervention effects and examine whether there may be a potential interaction between the baseline delayed gratification score and treatment arm.

Methods: Monitoring

Data and Safety Monitoring Board

A Data and Safety Monitoring Board is an independent group of experts convened to protect the safety of research subjects and to ensure that the scientific goals of the project are being met.
A Data and Safety Monitoring Board (DSMB) will be appointed by and act in an advisory capacity to the National Institute on Aging (NIA) to monitor participant safety, data quality and to evaluate the progress of the study.

DSMB Responsibilities

The DSMB will perform several duties:

1. They will review and approve the research protocol and plans for data and safety monitoring prior to the study.
2. They will evaluate the progress of the trial. This will include assessment of data quality, participant recruitment, accrual and retention, participant risk versus benefit, performance of trial sites, and study outcomes. This assessment will be performed at meetings every 6 months during the clinical trial and more frequently if needed.
3. They will make recommendations to ensure that all of the issues above are appropriately addressed. Study PIs will be responsible for responding to all recommendations of the DSMB and submitting DSMB reports to the IRB.

DSMB Membership and Affiliation

The DSMB will be composed of experts in clinical trials, medical economics, general internal medicine, and biostatistics, along with the project PI (Dr. Volpp), Dr. Yancy, and Dr. Troxel as non-voting members. Dr. Volpp will be responsible for maintaining communication between the DSMB and the individual project staff. We consider the proposed trial to be relatively low risk.

Therefore, we have arranged for a monitoring committee that is assigned to review the study and staff training protocols, monitor the trial for safety and adverse events, and conduct semi-annual meetings. These members will

not be involved directly with the trial. The members that we propose to serve on this committee and their activities are:

- **Philip Greenland, M.D.:** Dr. Greenland is the Harry W. Dingham Professor and Senior Associate Dean for Clinical and Translational Research at Northwestern University and Director of the Northwestern University Clinical and Translational Sciences (NUCATS) Institute. He is a well-known expert in the field of prevention of cardiovascular disease.
- **Michael K. Parides, Ph.D.:** Dr. Parides is Professor of Health Policy at the Mount Sinai School of Medicine, and Director of Biostatistics at the International Center for Health Outcomes and Innovation Research (InCHOIR) who specializes in clinical trials, linear models and analysis of categorical data, and has extensive experience in both conducting and monitoring randomized trials, especially in cardiology.
- **Allison Rosen, M.D., Sc.D., M.P.H.:** Dr. Rosen is an Associate Professor at the University of Massachusetts. She is an expert on evaluation of approaches to improving the value of U.S. health care spending.

Adverse Event Monitoring and Surveillance

The study will monitor the medical safety of participants. One aspect of this monitoring is to evaluate potential volunteers at screening to determine whether it is safe for them to participate in the planned intervention. Participants' weight loss will be monitored once they are enrolled in the trial. If a subject has lost too much weight too quickly, the safety of continuing or resuming participation in the study will be determined by the participant's PCP in conjunction with the study medical monitor and Principal Investigators. An alert will trigger to the clinical research coordinator if a participant has lost more than 10 pounds in one week or 20 pounds in a month.

Adverse Event Surveillance

According to the *Penn Manual for Clinical Research*, Adverse Events are events that:

- Are unanticipated ***--and--***
- Suggest that participants or others are at greater risk of harm than was

previously known or recognized.

This definition specifically includes the following:

- Information that indicates a change to the risks or potential benefits of the research, in terms of severity or frequency.
- Breach of confidentiality.
- Incarceration of a participant when the research was not previously approved under Subpart C and the investigator believes it is in the best interest of the subject to remain in the study.
- Events that require prompt reporting to the sponsor.
- Complaint of a participant when the complaint indicates unexpected risks or the complaint cannot be resolved by the research team.
- Protocol violation (meaning an accidental or unintentional change to the IRB approved protocol) that placed one or more participants at increased risk, or has the potential to occur again.
- Sponsor-imposed suspension

Unexpected/unanticipated

An event is classified as “unanticipated” when the specificity or severity is not reflected in the study documents including the protocol, or informed consent document.

Related to Study Procedures

The determination of how likely the event is related to the study procedures is made by the principal investigator, the classification of which may vary. The IRB asks if the event is "more likely than not" related to the study procedures.

Involved Risk to Participants or Others

"Participants or others" may involve anyone, including research subjects, research staff, or others not directly

involved in the research. The unanticipated problem can occur in either clinical or non-clinical research. "Risks" include physical, psychological, economic, legal or social consequences.

Defining Serious Adverse Events

According to the *Penn Manual for Clinical Research*, and as defined by the Food and Drug Administration, serious adverse events are defined by one of the following:

- Death
- Life-threatening experience
- Inpatient hospitalization or prolongation of hospitalization
- Persistent or significant disability/incapacity
- Congenital anomaly/birth defect in the subject's offspring
- An important medical event that, based upon appropriate medical judgment, may jeopardize the subject and may require medical or surgical intervention to prevent one of the outcomes listed above.
-

Periodic Surveillance of Adverse Events

Surveillance for SAEs and other relevant clinical events that may be associated with study participation will occur on an ongoing basis. The planned procedures for minimizing risk to subjects for potential side effects of the intervention involve careful screening prior to enrollment and careful monitoring after enrollment. In addition, estimates of the quantity of risk are given when data is available and the likely effectiveness of procedures to minimize risk is discussed when appropriate. In the event of an adverse effect necessitating medical or professional intervention, referral to the subject’s primary care provider or an appropriate specialist will occur.

Adverse Event Reporting

Safety-related events will be reported in a timely fashion as required by the Data and Safety Monitoring Board, the local IRB, and NIA. The study principal investigators will be directly responsible for identifying and reporting all serious adverse events, protocol deviations/violations and unanticipated events to the IRBs and funding agencies

promptly, as appropriate. Additionally, the DSMB will receive monthly reports of SAEs.

Study Procedures

Procedures

Potentially eligible patients will be sent emails directing them to the Way to Health website. Participants will be required to sign up online and complete the screening questionnaire where they will be asked to document their previous weight loss. The screening questionnaire has been attached.

Participants will be asked to electronically sign the consent and complete the demographic survey on the platform. Participants will then be randomized to one of two study arms. Immediately following randomization, each participant will receive a description of her assigned treatment condition by email and on the study websites dashboard (See Control and Escalating Lottery dashboard instructions attached). Participants will self-monitor their weights daily. Every day, at a time convenient for the participant, participants will weigh themselves using the internet-enabled scale. The scale will wirelessly transmit their weight data where it will be stored on the Way to Health portal. Each week, subjects will be text paged with feedback on their progress relative to their weekly goal. Participants in the escalating lottery arm will be eligible for a weekly lottery with an expected value of $3.98 in week 1 that will increase by $0.43 per week if, prior to resolution of the lottery, they weighed in 6 out of 7 days the prior week. If participants in the escalating rewards arm miss the target in a given week, they will go back to the beginning of the escalating rewards schedule, with one exception: participants will be given the opportunity to return to their previous place in the schedule if they meet goals the following week.

The lottery provides both infrequent large payoffs (a 1 in 100 chance at a $110 reward in week 1) and frequent small payoffs (an 18 in 100 chance at a $16 reward in the week 1) since subjects are motivated by both the future (fixation on large potential winnings) and the past (how often did I win?). Participants in the lottery arm will be asked to pick a 2-digit number upon entry into the study, e.g. 27. A two digit random number will be generated for each week. If the random number includes one digit, either a 2 or 7 in the same position as the

participant's number (an 18 in 100 chance), and the subject weighed in at least 6 days that week, s/ he wins a small reward. If the random number matches 27 exactly (a 1 in 100 chance) the subject wins the large reward. When a subject does not meet their goal of weighing in 6/7 days in a week, we will still generate a 2-digit daily number and tell the subject if s/he would have won the lottery that week if s/he had weighed in at least 6 out of 7 days. We believe that a desire to avoid the regret associated with not winning combined with learning that one would have won, had one been adherent, will motivate participants to a greater degree than only on the value of the rewards. Other than the total number of lottery weeks (24), there is no limit to the number of times a person could win the daily lottery if their number comes up; however, for participants who make all of their weekly goals for the entire 6 months (24 weeks) of the intervention, the expected value of the lottery is approximately $214.

All participants will be required to complete online surveys at months 6 and 12. Participants will receive automated emails/text messages from the Way to Health platform notifying them of their compensation payment for completing each of the required surveys at months 6 an 12. Participants will receive $50 for surveys at months 6 and 12. All payments will be approved electronically from the Way to Health platform. Payment information will be sent to Wells Fargo to process and send to participants via check every week. Between months 7-12 (Phase II), participants will not receive incentives tied to weighing in to evaluate sustainability of effects achieved in Phase I.

Subject Privacy

Potential participants will be identified by reviewing Weight Watchers records using the inclusion criteria. Weight Watchers will facilitate communication to these members using existing communication channels (email). Interested participants will be directed to the Way to Health portal where they will be asked to enter data related to eligibility and their demographic characteristics. Enrollment will include a description of the voluntary nature of participation, the study procedures, risks and potential benefits in detail. The enrollment procedure will provide the opportunity for potential participants to ask questions and review the consent form information with family and friends prior to making a decision to participate. Participants will be told that they do not have to answer any questions if they do not wish and can drop out of the study at any time, without affecting their medical care or the

cost of their care. They will be told that they may not benefit directly from the study and that all information will be kept strictly confidential, except as required by law. Subjects will be given a copy of the consent document. All efforts will be made by study staff to ensure subject privacy.

Consent

Consent Process

Overview

Potential participants will be identified by reviewing Weight Watchers records using our eligibility criteria. Weight Watchers will facilitate communication to these members using existing communication channels (email). Potentially eligible individuals will be invited to visit the WTH portal. Potentially eligible individuals will enroll directly through the WTH platform, which steps them through screening and consent process electronically.

Risk / Benefit

Potential Study Risks

The incentive programs could be detrimental to individuals with certain psychiatric issues such as bulimia nervosa or gambling disorder. We will screen for and exclude people with these disorders during recruitment. Because of the low prevalence of these disorders in our target population, we anticipate this to be an infrequent occurrence. The main risk is loss of confidentiality, which will be protected as described below. Database Security/Protection against Risk. To assure that patient, physician and other informant confidentiality is preserved, individual identifiers (such as name and medical record number/physician billing identifier) are stored in a single password protected system that is accessible only to study research, analysis and IT staff.

This system is hosted on site at The University of Pennsylvania (UPenn) and is protected by a secure firewall. Once a participant is in this system, they will be given a unique study identification number (ID). Any datasets and computer files that leave the firewall will be stripped of all identifiers and individuals will be referred to by their study ID. The study ID will also be used on all analytical files. Additionally, any information that leaves this system to communicate with third party data sources (biometrics devices, survey software, etc.) will be stripped of any identifiers and transmitted in encrypted format. The same unique study ID will be used to link these outside data to the participants. Social security numbers, bank account and routing numbers for all participants to whom payments are sent will also be transmitted in encrypted format to UPenn's Financial Systems/Comptrollers Department where data will be stored for compliance with W-9 form reporting requirements. After the social security numbers are no longer needed they will be deleted from our system. The Way to Health (WTH) Research Data Center staff is responsible for preventing unauthorized access to the trial participant tracking system database. It is important to note that the Way to Health database server and individual study databases have never been compromised as a result of the extremely rigorous and secure network firewall technologies. The secure servers are located in a specially designed, highly secured facility at UPenn with dedicated uninterrupted power supply and strictly limited access. The study will utilize a client-server deployed Data Management System (DMS) rather than a 'Store and Forward' database configuration, obviating research site database security concerns. Confidential participant information will be entered into the database. Thereafter, confidential information will be made available to authorized users only as specifically needed. No one can gain access to an individual MySQL database table unless explicitly granted a user ID, password, and specific access. Even those with user names and passwords cannot gain access to the tables that contain the identifying participant information. No results will be reported in a personally identifiable manner. All tracking system data will be password-protected with several levels of protection. The first will allow access to the operating system of the computer. The second will allow access to the basic menus of the integrated system; within certain menu options, such as database browsing, a third password will be required. Our prior research employing similar precautions has demonstrated that these techniques are very successful in assuring the protection of subjects. The same procedure used for the analysis of automated data sources to ensure protection of patient information will be used for the survey data, in that patient identifiers will be used only for linkage purposes or to contact patients. The study identification number, and no other identifying information, will be used on all data

collection instruments. All study staff will be reminded to appreciate the confidential nature of the data collected and contained in these databases. In order to monitor for excessive weight loss, the CRC will be sent an alert if a participant loses more than 10pounds in one week and/or 20 pounds in one month. In all such instances the participant will be contacted to screen for unsafe weight loss behaviors. The PIs will review all such cases.

Potential Study Benefits

This study proposes to focus on the effectiveness of financial incentives as a means of encouraging weight maintenance in obese volunteers. Given the lack of effective approaches to maintenance of weight loss, the proposed study will contribute to the field by examining the effectiveness of an innovative incentive design that involves study-provided incentives for frequent weighing of oneself and its possible effects on weight loss maintenance. This type of intervention could help ameliorate disparities in health outcomes because obesity is more prevalent in minority populations and is a major risk factor for adverse health events. This approach has potentially broad generalizability in treating the obesity epidemic nationally, as these types of financial incentives could be set up by insurers and broadly utilized. As stated previously, findings from this important research will provide further information concerning the management of obesity and maintenance of weight loss.

Dissemination

Results of the study will be made public using clinicaltrials.gov, manuscript publication, and presentations at health conferences. The policy implications of this work call for a dissemination strategy that goes beyond presentations at health professional meetings and publication in scholarly journals. Our dissemination activities are designed to reach specific audiences of leading figures in health care delivery with information they can use, and we will utilize the expertise of the Advisory Board of the LDI CHIBE, which was designed with this in mind.

Appendix: Informed Consent Form

UNIVERSITY OF PENNSYLVANIA RESEARCH SUBJECT INFORMED CONSENT & HIPAA AUTHORIZATION FORM

Protocol Title: A randomized trial of financial incentives for maintenance of weight loss part II

Principal Investigators: Kevin Volpp, MD, PhD/ William Yancy, MD, MHSc

Contact: 215-746-8439

Why am I being asked to volunteer?

You are being invited to participate in a research study. You are being invited because you meet the initial requirements for enrollment in this study, including that you have recently lost about 11 pounds of weight in the past 4-6 months and have an online Weight Watchers membership. Your participation in this study is voluntary, which means you can choose whether or not you want to participate. Before you can make your decision, you will need to know what the study is about, the possible risks and benefits of being in this study, and what you will be asked to do in this study. The following screens will explain the study in detail. After reviewing this information, you will see an option to click links to participate in the study or to not participate. If you choose to participate in this study, you may withdraw at any time.

What is the purpose of this research study?

The purpose of the study is to learn more about effective ways to maintain weight loss in adults. We will test different strategies for helping people maintain weight loss.

How long will I be in the study? How many people will be in the study?

The study will last 52 weeks (12 months). You will be one of 258 people in the study.

What am I being asked to do?

During the enrollment process, you will complete an online questionnaire that will ask you some questions about your age, gender and health. You do not have to answer any questions which make you feel uncomfortable. This online survey will take about 10 minutes.

You will be "randomized" into one of 2 research study groups. Randomization means that you are put into a group by chance. A computer program will place you in one of the research groups. Neither you nor the researchers can choose the group you will be in.

For 12 months, participants in each of the 2 groups will weigh themselves daily at home with a wireless scale that we will provide to you at no charge. You will be asked to weigh in daily, striving to weigh in at least 6 out of 7 days per week.

You will have a personalized web portal that you will log into that will allow you to track your weight, and your progress towards meeting your weight loss goals. The study website will also send you weekly messages regarding your goals.

At the end of months 6 and 12, you will be asked to complete an online survey that will take approximately 20 minutes to complete.

What are the possible risks or discomforts?

If you lose weight rapidly, there are potential medical risks to very rapid weight loss. Risks of rapid weight loss include: gallstones (signs of gallstones can include pain in your upper right abdomen, nausea and vomiting, fever, or changes in the color of your urine or stool), constipation, diarrhea, hair loss, loss of electrolytes that can lead to heart arrhythmias, malnourishment and feeling too cold. Study staff will monitor your weight and notify the study clinician if you have lost a large amount of weight rapidly. In this case, the study team may ask you to talk to your primary care doctor.

In addition, it is possible that if you do not meet your weekly weighing goal you may experience some disappointment.

You cannot be in this study if you are pregnant or if you become pregnant during the study. This is because weight loss is generally not recommended during pregnancy.

In addition, although the study takes a number of steps to prevent risks of participation, it is always possible that there are additional risks that we are unaware of. If we learn about additional risks during the study, we will tell you about them.

Study staff will be available to discuss any concerns you may have about your participation in the study. Contact information for these individuals is listed at the beginning of this consent form.. If for any reason you are not comfortable discussing your concerns with study staff directly, the staff can help you find appropriate contact information for other counseling services

provided by your health insurance.

What if new information becomes available about the study?

During the course of this study, we may find out information that could be important to you. We will notify you as soon as possible if such information becomes available. You will always have the right to change your mind about being in this study.

What are the possible benefits of the study?

Participation in this study may help you to lose weight or maintain weight you have already lost, which could improve your health and reduce your risk of diseases such as diabetes, heart disease, arthritis, and certain kinds of cancer. If this program is effective, it could help other people lose weight.

What other choices do I have if I do not participate?

You can choose not be in the study.

If you choose not to be in the study you may always consult with your primary care doctor and/or your Weight Watchers program leader regarding appropriate weight loss and maintenance strategies as well as other programs that are available.

Will I be paid for being in this study?

Everyone in this study will have the opportunity to earn a total of $100 for completing online surveys at months 6 and 12. In addition, some participants may earn additional money if they successfully weigh in 6 out of 7 days each week.

In order to be paid for participating in this study, you will be asked to provide your Social Security number. The University of Pennsylvania requires that we collect Social Security numbers for all research participants who get paid for being in a research study. You do not have to provide your Social Security number, but if you choose not to, you will not be able to participate in the study.

Payment received as compensation for participation in research is considered taxable income to the research subject. If payment to an individual is $600 or more in any one calendar year, the University of Pennsylvania is required to report this information to the Internal Revenue Service (IRS). Research participant payments of $600 or more during any calendar year will result in a 1099 (Miscellaneous Income) form being issued to the individual and a copy sent to the IRS.

Will I have to pay for anything?

If you choose to receive messages from Way to Health by text message you will be responsible for the costs associated with the receipt of these messages. For example, if you have a monthly text messaging plan, these messages will count towards your monthly text messaging total. If

you do not have a plan, you will be charged the standard text messaging fees by your wireless provider. You may receive a maximum of 35 text messages from Way to Health each month. You can edit your Way to Health profile or contact the study coordinator at any point during the study to change your notification preferences. If you would rather receive messages via email, you can select this preference at any time.

When is the Study over? Can I leave the Study before it ends?

The study is expected to end after all participants have completed all visits and all the information has been collected. The planned duration for each individual is 52 weeks (12 months). Your participation in the study may be terminated without your consent for the following reasons:

- The Principal Investigator feels it is best for your safety and/or health. If this is the reason for termination you will be informed of the reasons why.
- You have not followed the study instructions.
- The Principal Investigator, the sponsor, or the Office of Regulatory Affairs at the University of Pennsylvania can stop the study anytime.

You have the right to drop out of the research study at any time during your participation. There is no penalty or loss of benefits to which you are otherwise entitled if you decide to do so. Withdrawal will not interfere with your future health care or your relationship with Weight Watchers.

If you no longer wish to be in the research study, please contact the study Project Manager, Tori Hilbert by phone at 215-746-8439 or by email at ulrichv@mail.med.upenn.edu and take the following steps:

- Discuss your reasons for leaving the study with the Study Project Manager.
- Provide a brief written statement indicating that you would like to exit the study.

There are no negative consequences for leaving the study early.

Use of Study Materials

Information about you that is collected during the study will not be given to others (unless it is required by a government agency or other legal authority). This means that no one (not your family, your doctor, your insurance company, or your employer) will have access to this information during the study.

Who can see or use my information? How will my personal information be protected?

The Principal Investigators (Kevin Volpp, MD, PhD, William Yancy, MD, MHSc) and staff involved with the study will keep your personal information strictly confidential. It will be kept in a

secured, password-protected file at the University of Pennsylvania. At any time, you may ask to see your personal information (such as name and address) and correct it if necessary.

What information about me may be collected or shared with others?

Weight Watchers will provide your weight information to the University of Pennsylvania to support the study. In addition, information about your weights and weight loss activities during the study will be combined with information from other people in the study. This information will be used to learn about which methods of encouraging weight loss and maintenance are most effective.

We will collect information about your:

- Name, address, email address, social security number, and telephone number
- Weight, height
- Personal characteristics such as age, gender, and income
- Dietary habits and level of physical activity
- Weight Watchers member ID number

This personal information will not be shared with your healthcare providers, your insurance company, or your employer. When you sign this consent form, you agree to have your personal and medical information used as described here.

We may post positive feedback you give the study team anonymously (without using your name) on the University of Pennsylvania Way to Health marketing website. Additionally, we may use the anonymous feedback in presentations.

Who, outside of the University of Pennsylvania, might receive my information?

- Wells Fargo (We will share your Social Security Number, Name, and Address with Wells Fargo in order to coordinate your payments)
- Withings (to record your weight from the wireless scale. Withings may also ask you to provide your name and date of birth to create an account, but we will not be collecting this information). You can access privacy information on Withings website: http://www.withings.com/en/wirelessscale/faq:
- Twilio Cloud Communication (We will only provide your phone number to send you text messages. You will only receive study related text messages)
- Qualtrics (We won't be sharing any of your information with Qualtrics. We will only be interacting with Qualtrics to use their online survey service and to collect your answers to survey questions)
- Weight Watchers (to facilitate their provision of weight information to us)

- Penn Approved Data Center

Why is my information being collected, and how will it be used?

Your personal information will be used by the research team to contact you during the study. Your responses to questionnaires and results of weigh-ins will be used to:

- Do the research
- Oversee the research
- Make sure the research was done correctly
- Ensure that money for your participation in the research is sent to you

How long may the University of Pennsylvania use or disclose my personal health information?

Your authorization for use of your personal health information for this specific study does not expire.

Your information will be held in the research database. However, the University of Pennsylvania may not re-use or disclose information collected in this study for a purpose other than this study unless:

- You have given written authorization
- The University of Pennsylvania Institutional Review Board grants permission
- As permitted by law

Can I change my mind about giving permission for use of my information?

Yes. You may withdraw or take away your permission to use and disclose your health information at any time. You do this by sending written notice to the study PI. If you withdraw your permission, you will not be able to stay in this study.

What if I decide not to give permission to use and give out my health information?

Then you will not be able to be in this research study. You will be given a copy of this Informed Consent and Research Subject HIPAA Authorization describing your confidentiality and privacy rights for this study. By signing this document you are permitting the University of Pennsylvania to use and disclose personal health information collected about you for research purposes as described above.

Who can I call with questions, complaints or if I'm concerned about my rights as a

research subject?

If you have questions, concerns or complaints regarding your participation in this research study or if you have any questions about your rights as a research subject, you should speak with the Project Manager listed on Page 4 or the Principal Investigators listed on Page 1 of this form. If a member of the research team cannot be reached or you want to talk to someone other than those working on the study, you may contact the Office of Regulatory Affairs at the University of Pennsylvania with any questions, concerns or complaints by calling (215) 898-2614.

Next steps

When you click the "I want to participate" button below, you are agreeing to take part in this research study. This means that you have read the consent form, your questions have been answered, and you have decided to volunteer. Clicking the "I want to participate" button also means that you are permitting the University of Pennsylvania to use personal health information collected about you for research purposes within our institution.
