# Supplementary material for: Effect of Escalating Financial Incentive Rewards on Maintenance of Weight Loss: A Randomized Clinical Trial
Source: JAMA Netw Open. 2019 Nov 1;2(11):e1914393. doi: 10.1001/jamanetworkopen.2019.14393 (PMC6826643; doi:10.1001/jamanetworkopen.2019.14393)
Supplement: Supplement 2. — Data Sharing Statement [file jamanetwopen-2-e1914393-s002.pdf]

Data Sharing Statement

Yancy, Jr. Effect of Escalating Financial Incentive Rewards on Maintenance of Weight Loss. *JAMA Netw Open*. Published November 01, 2019. 10.1001/jamanetworkopen.2019.14393

Data
Data available: No
